# Factors associated with poor sleep health in adults at risk for obstructive sleep apnea

**DOI:** 10.1007/s11325-026-03621-2

**Published:** 2026-03-03

**Authors:** Jaqueline M. Pereira, Ricardo L. M. Duarte, Keren Cozer, Ana Paula D. Fernandes, Luciane F. Mello, Fernanda C. Q. Mello, David Gozal

**Affiliations:** 1 Hospital Universitário Clementino Fraga Filho - Universidade Federal Do Rio de Janeiro, Rio de Janeiro, Brazil; 2 Instituto de Doenças Do Tórax - Universidade Federal Do Rio de Janeiro, Rua Professor Rodolpho Paulo Rocco, 255–1° andar – sala 01D 58/60, Rio de Janeiro, CEP 21941-913 Brazil; 3 Departments of Pediatrics and Biomedical Sciences, Joan C. Edwards School of Medicine, Marshall University, Huntington, WV USA

**Keywords:** Sleep quality, Ru-SATED, Insomnia, Obstructive sleep apnea, COMISA

## Abstract

**Introduction:**

Sleep quality is essential for health, yet few studies have assessed this issue in adults with suspected obstructive sleep apnea (OSA). This study aimed to evaluate sleep health in these adults using the previously validated Ru-SATED questionnaire during home sleep apnea testing.

**Methods:**

A cross-sectional study was conducted with adults who completed the multidimensional Ru-SATED questionnaire, which evaluates six main dimensions of sleep health. Participants were divided into two groups based on their Ru-SATED scores: good sleep health (≥ 8 points) and poor sleep health (< 8 points). Correlation was assessed using Spearman's coefficient (r). Logistic regression tests were performed to identify predictors of poor sleep quality.

**Results:**

A total of 415 patients were evaluated: 220 patients (53.0%) reported good sleep quality, while 195 patients (47.0%) experienced poor sleep quality. Ru-SATED scores were correlated with the severity of insomnia, as measured by the Insomnia Severity Index [ISI] (*r* = -0.625; *p* < 0.001), but not with the respiratory event index [REI] (*r* = -0.076; *p* = 0.123). In the univariate analysis, four parameters emerged as significant predictors of poor sleep health: excessive daytime sleepiness (*p* = 0.008), insomnia (*p* < 0.001), ISI (*p* < 0.001), and REI (*p* = 0.020). In the multivariate analysis, only ISI values emerged as an independent predictor of poor sleep quality (adjusted odds ratio: 1.219; 95% confidence interval: 1.154–1.287; *p* < 0.001).

**Conclusion:**

Among individuals with clinically suspected OSA, a high prevalence of poor sleep health was detected and strongly associated with the severity of insomnia.

## Introduction

Quality sleep is essential for the well-being of children, adolescents, and adults. It plays a crucial role in cognitive functioning as well as in mental, cardiovascular, cerebrovascular, and metabolic health [1]. Recent studies have revealed associations between overall sleep health scores and a large array of outcomes, including mortality, cognitive decline, depression, and healthcare costs [2–5]. Maintaining healthy sleep is highly important for public safety, as it is estimated that one-third of motor vehicle crashes and injuries are linked to sleep deprivation and fatigue [6]. Furthermore, poor sleep can lead to increased job absenteeism and reduced productivity, and may negatively impact intellectual performance and physical health [7].

The Pittsburgh Sleep Quality Index (PSQI) is one of the most widely validated tools for assessing subjective sleep quality. It consists of 19 questions that represent seven components of sleep quality: subjective sleep quality, sleep latency, sleep duration, sleep efficiency, sleep disturbance, intake of sleep medication, and daytime impacts [8]. In contrast, the Ru-SATED questionnaire is a practical and concise self-reported tool that evaluates six key dimensions of sleep health: regularity, satisfaction, daytime alertness, timing, efficiency, and duration of sleep [8–11]. Each item on the Ru-SATED is scored from 0 to 2, resulting in a final score ranging from 0 to 12, where higher scores indicate better subjective sleep quality [10]. Several studies have suggested that the multidimensional Ru-SATED is a valid measure for assessing subjective sleep quality [12–15]. This instrument is easy to complete, which can be valuable in daily clinical practice. Additionally, research has linked all dimensions of the Ru-SATED framework to adverse health outcomes [8].

Obstructive sleep apnea (OSA) is the most common sleep-disordered breathing (SDB) condition, characterized by the collapse of the upper airway during sleep, leading to intermittent hypoxemia, disrupted sleep, and increased respiratory effort [16]. OSA is associated with various negative health outcomes and significant medical costs [17]. In turn, insomnia can result in reduced quality of life, increased psychiatric comorbidities, work-related difficulties, and increased cardiovascular risk [18, 19]. Unlike OSA, which requires an objective sleep study for diagnosis, insomnia is established based on clinical symptoms. Of note, patients suffering from comorbid insomnia and OSA (COMISA) often experience poorer sleep quality, worse daytime functioning, increased cardometabolic morbidity, and lower quality of life compared to those with only OSA or insomnia [20–24].

Individuals with sleep disorders often report poor sleep quality, and the Ru-SATED instrument is a simple tool that can be easily used during routine clinical evaluations. However, surprisingly few studies have evaluated the use of the Ru-SATED tool in adults with suspected SDB referred for an objective diagnostic sleep study. Furthermore, although this questionnaire has been validated in Portuguese [25], we are unaware of any studies in Brazil focused on patients with sleep disorders. Therefore, our main objective was to evaluate the prevalence and factors associated with the presence of poor sleep health using the Ru-SATED questionnaire in adults with suspected OSA who underwent a home sleep apnea test (HSAT).

## Methods

The study protocol complied with the Declaration of Helsinki and received prior approval from the Ethics Committee of the *Universidade Federal do Rio de Janeiro* (CAAE: 64135922.2.0000.5257). All participants provided written informed consent before enrolling in the study, and their anonymity was strictly maintained throughout the process. Additionally, this study adhered closely to the STROBE checklist guidelines [26].

### Study design

From June 2023 to June 2025, a cross-sectional study was conducted at the *Hospital Universitário Clementino Fraga Filho*, at the *Universidade Federal do Rio de Janeiro*. Individuals were referred for a sleep study by their primary care physicians due to suspected OSA. The authors did not participate in the enrollment of study participants. Subsequently, eligible patients were invited to participate in the study consecutively to minimize selection bias. All participants were asked to complete the six items of the Ru-SATED questionnaire. Following this, each participant underwent a HSAT using the ApneaLink Air™ device (ResMed, Sydney, Australia). The test was performed in the participants' home environments after they received thorough instructions on how to use the device. The inclusion criteria for this study were adult individuals aged 18 years or older who were suspected of having OSA and were undergoing HSAT. Conversely, subjects were excluded from the study for any of the following reasons: a previous diagnosis of OSA with continuous positive airway pressure treatment, a diagnosis of central sleep apnea, a technically inadequate HSAT (total recording time of less than 240 min), incomplete responses to the Ru-SATED questionnaire, nocturnal home oxygen therapy, or failure to sign the informed consent form.

### Data collection

All clinical, demographic, and anthropometric data were systematically collected by a single researcher (J.M.P.). Body mass index (BMI) was calculated by dividing weight (in kilograms) by height (in meters) squared (kg/m^2^). Neck and waist circumferences were measured in centimeters using a measuring tape. When measuring neck circumference, the patient sat with their head positioned in the Frankfurt horizontal plane at the level of the laryngeal prominence. For waist circumference, the patient stood upright, and the measurement was taken at the midpoint between the lower rib cage and the iliac crest. The Epworth Sleepiness Scale (ESS) is a questionnaire that subjectively assesses excessive daytime sleepiness (EDS) through 8 questions, each scored from 0 (no chance of dozing) to 3 (high chance of dozing). A score of 11 or higher (out of a possible 24) indicates the presence of EDS [27].

The clinical diagnosis of chronic insomnia was characterized by the presence of one or more of the following nighttime complaints: 1) Difficulty initiating sleep; 2) Waking up during the night and having trouble falling back asleep; 3) Waking up earlier than desired and struggling to return to sleep. These complaints must occur for at least three nights per week over three months or longer and should be associated with daytime impairments, such as fatigue, difficulties with concentration or memory, issues in social or occupational functioning, mood changes, daytime sleepiness, and/or non-restorative sleep [19]. The severity of insomnia was assessed using the Insomnia Severity Index (ISI), a 7-item questionnaire that subjectively evaluates the intensity of both nighttime and daytime insomnia symptoms. Higher scores on this index indicate a greater severity of insomnia [28].

### Ru-SATED instrument

Multidimensional self-reported sleep health was systematically measured using the Ru-SATED questionnaire [8], which consists of a self-administered questionnaire that assesses six dimensions of sleep health: “Sleep regularity” (Do you go to bed and get up at more or less the same time every day?), “Sleep satisfaction” (Are you satisfied with your sleep?), “Alertness” (Can you stay awake all day without taking a nap?), “Timing” (Are you asleep [or in bed] between 2 and 4 a.m.?), “Efficiency” (Do you spend less than 30 min awake at night), and “Duration” (Do you sleep between 6 and 8 h/day?). The items were assessed on a 3-point Likert frequency scale (0: “Never or rarely”; 1: “Sometimes”; and 2: “Often or always”). The total score, which is obtained by adding the item scores, ranges from 0 to 12, with higher scores indicating better sleep quality [10, 11]. Study participants were categorized based on their Ru-SATED scores into two independent subgroups: those with good sleep health (Ru-SATED ≥ 8 points) and those with poor sleep health (Ru-SATED < 8 points), following previously published guidelines [10].

### Sleep test

The HSAT involves continuous monitoring of airflow using a nasal cannula, a chest impedance belt, and a pulse oximeter that tracks both oximetric data and heart rate. All polygraphic recordings were manually interpreted by a certified sleep medicine physician (R.L.M.D.) in accordance with guidelines published by the American Academy of Sleep Medicine [29]. HSAT was scored without prior knowledge of the clinical measurements, including the Ru-SATED scores, which were assessed by another researcher (J.M.P.). Apneas were classified as a ≥ 90% drop in airflow from the baseline, lasting at least 10 s, while hypopneas were classified as a ≥ 30% drop from the pre-event baseline for ≥ 10 s, associated with an oxygen desaturation of ≥ 4% [29]. The respiratory event index (REI) is defined as the sum of apneas and hypopneas divided by the total recording time (in hours). SDB was categorized as either OSA, where obstructive events predominated, or central sleep apnea, where central respiratory events predominated over obstructive events. The diagnosis of OSA was based on an REI of ≥ 5.0 events per hour, with a predominance of obstructive respiratory events [29].

### Statistical analysis

Statistical analysis of the data was conducted using the SPSS for Windows program (version 21.0; SPSS; Chicago, IL, USA). Results are presented as median and interquartile range (IQR) for continuous variables, while categorical variables are expressed as frequency (n) and corresponding percentage (%). Group comparisons for categorical variables were made using the chi-square test. For numerical variables, the non-parametric Mann–Whitney or Kruskal–Wallis tests were utilized. The correlation between numerical variables was assessed using Spearman's correlation coefficient (r). Multiple logistic regression analyses were systematically employed to identify factors associated with poor sleep quality (the dependent variable), as evaluated by the Ru-SATED questionnaire. This included statistically relevant variables based on previous univariate analysis (*p* < 0.05). The calibration of the logistic regression model was evaluated using the Hosmer–Lemeshow chi-squared test, with a *p*-value under 0.05 indicating poor calibration. All results are presented as crude and adjusted odds ratios (ORs) with 95% confidence intervals (CIs) derived from univariate or multivariate analyses, respectively. A two-tailed *p*-value of less than 0.05 was considered statistically significant.

## Results

A total of 445 subjects with clinically suspected SDB were consecutively recruited after being referred for HSAT by their primary care physicians. Thirty patients were excluded for the following reasons: 28 due to incomplete clinical, demographic, and/or anthropometric data, and 2 because of technically inadequate HSAT, and their unwillingness to repeat the test later. As a result, 415 patients were evaluated, comprising 241 women (58.1%) and 174 men (41.9%). The clinical, demographic, and anthropometric variables, along with the data obtained from HSAT, are presented in Table 1. The median age of the participants was 53.0 years (IQR: 37.0–65.0), and the median BMI was 30.0 kg/m^2^ (IQR: 25.6–34.6). Among the participants, 201 patients (48.4%) experienced EDS, while 212 (51.1%) had hypertension. According to the Ru-SATED questionnaire, 220 subjects (53.0%) reported good sleep health, while 195 (47.0%) reported poor sleep health. Poor quality sleepers reported a statistically higher prevalence of EDS (*p* = 0.008), chronic insomnia, and higher values of ISI (both with *p* < 0.001) compared to good quality sleepers. Otherwise, no significant differences were found in the parameters obtained from HSAT between the groups categorized by subjective sleep health (all *p*-values > 0.05).

**Table 1:** Summary of the participant characteristics

Parameters	Total *n* = 415	Good sleepers *n* = 220	Poor sleepers *n* = 195	*p*-value
Clinical data				
Female sex	241 (58.1)	132 (60.0)	109 (55.9)	0.426
Age, years	53.0 (37.0–65.0)	52.0 (37.0–65.0)	54.0 (36.0–65.0)	0.883
BMI, kg/m^2^	30.0 (25.6–34.6)	29.0 (25.6–34.1)	30.4 (24.9–35.1)	0.317
NC, cm	39.0 (36.0–42.0)	38.0 (36.0–42.0)	39.0 (36.0–43.0)	0.135
WC, cm	101.5 (91.0–112.0)	100.0 (91.0–111.0)	91.0 (103.5–113.0)	0.338
EDS	201 (48.4)	93 (42.3)	108 (55.4)	0.008
Hypertension	212 (51.1)	105 (47.7)	107 (54.9)	0.168
Type 2 DM	110 (26.5)	52 (23.6)	58 (29.7)	0.181
Dyslipidemia	102 (24.6)	51 (23.2)	51 (26.2)	0.495
Current smoking	30 (7.2)	16 (7.3)	14 (7.2)	0.999
Chronic insomnia	183 (44.1)	58 (26.4)	125 (64.1)	< 0.001
ISI, points	12.0 (7.0–17.0)	8.5 (4.0–13.0)	16.0 (12.0–20.0)	< 0.001
Ru-SATED, points	8.0 (6.0–10.0)	9.0 (8.0–10.7)	6.0 (4.0–6.0)	< 0.001
HSAT data				
REI, n/h	9.0 (5.0–19.8)	8.6 (5.2–17.7)	9.0 (5.0–24.0)	0.402
Baseline SpO_2_, %	96.0 (95.0–98.0)	96.0 (95.0–98.0)	96.0 (95.0–97.0)	0.468
Average SpO_2_, %	94.0 (91.0–95.0)	94.0 (92.0–95.0)	93.0 (91.0–95.0)	0.053
Nadir SpO_2_, %	82.0 (76.0–87.0)	82.0 (77.0–87.0)	82.0 (75.0–87.0)	0.434
T90, min	19.0 (2.0–87.0)	18.0 (2.0–77.0)	20.0 (2.0–96.0)	0.571
T90, %	6.0 (1.0–26.5)	6.0 (1.0–23.0)	7.0 (1.0–32.0)	0.426
ODI, n/h	6.4 (2.6–15.0)	6.6 (2.4–14.5)	6.3 (2.7–16.0)	0.487
OSA	316 (76.1)	169 (76.8)	147 (75.4)	0.818

Numerical and categorical variables were expressed as the median (interquartile range) and n (%). *BMI* body mass index; *NC* neck circumference; *WC* waist circumference; *DM* diabetes mellitus; *HSAT* home sleep apnea test; *REI* respiratory event index; *SpO*_2_ peripheral oxygen saturation; *T90* time with SpO_2_ < 90%; *ODI* oxygen desaturation index at 3%; *OSA* obstructive sleep apnea. Multidimensional sleep health was categorized according to Ru-SATED scores into good sleep health (≥ 8 points) and poor sleep health (< 8 points)

Table 2 reports the distribution of the six components of the Ru-SATED questionnaire across the entire cohort, distinctly categorizing individuals based on their sleep quality, i.e., good or poor. All 6 categories of the multicomponent Ru-SATED questionnaire were scored lower in individuals reporting poor sleep health than in those with good sleep health. In terms of "Satisfaction," only 4.1% of patients with poor sleep quality reported that they were often or always satisfied with their sleep. In comparison, 37.3% of patients with good sleep quality indicated that they were often or always satisfied with their sleep.

**Table 2:** Distribution of the Ru-SATED components in the cohort

Components	Total *n* = 415	Good sleepers *n* = 220	Poor sleepers *n* = 195
Regularity (R)			
Never or rarely (0)	56 (13.5)	7 (3.2)	49 (25.1)
Sometimes (1)	86 (20.7)	33 (15.0)	53 (27.2)
Often or always (2)	273 (65.8)	180 (81.8)	93 (47.7)
Satisfaction (S)			
Never or rarely (0)	210 (50.6)	55 (25.0)	155 (79.5)
Sometimes (1)	115 (27.7)	83 (37.7)	32 (16.4)
Often or always (2)	90 (21.7)	82 (37.3)	8 (4.1)
Alertness (A)			
Never or rarely (0)	97 (23.4)	27 (12.2)	70 (35.9)
Sometimes (1)	108 (26.0)	45 (20.5)	63 (32.3)
Often or always (2)	210 (50.6)	148 (67.3)	62 (31.8)
Timing (T)			
Never or rarely (0)	35 (8.4)	4 (1.8)	31 (15.9)
Sometimes (1)	120 (28.9)	40 (18.2)	80 (41.0)
Often or always (2)	260 (62.7)	176 (80.0)	84 (43.1)
Efficiency (E)			
Never or rarely (0)	123 (29.6)	28 (12.7)	95 (48.7)
Sometimes (1)	93 (22.4)	42 (19.1)	51 (26.2)
Often or always (2)	199 (48.0)	150 (68.2)	49 (25.1)
Duration (D)			
Never or rarely (0)	105 (25.3)	13 (5.9)	92 (47.2)
Sometimes (1)	74 (17.8)	26 (11.8)	48 (24.6)
Often or always (2)	236 (56.9)	181 (82.3)	55 (28.2)

Categorical variables were expressed as n (%). Sleep quality was measured using the Ru-SATED questionnaire that assesses six dimensions of sleep health: R (regularity), S (Satisfaction), A (Alertness), T (Timing), E (Efficiency), and D (Duration). The items were assessed on a 3-point Likert frequency scale: 0 (Never or rarely), 1 (Sometimes), and 2 (Often or always). Multidimensional sleep health was categorized according to Ru-SATED scores into good sleep health (≥ 8 points) and poor sleep health (< 8 points)

**Fig. 1:**
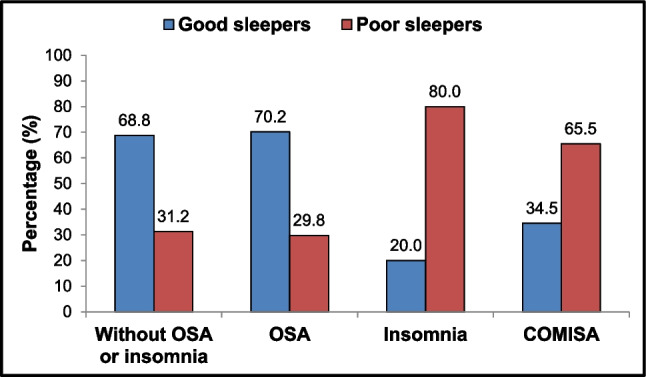
Distribution of study participants (*n* = 415) when categorized as good or poor sleepers across four groups: 64 subjects without obstructive sleep apnea (OSA) and insomnia, 168 with OSA only, 35 with insomnia only, and 148 with comorbid insomnia and OSA (COMISA). The results showed a higher percentage of poor sleepers in the insomnia and COMISA groups, while the control and OSA groups predominantly consisted of good sleepers. Multidimensional sleep health was categorized by Ru-SATED scores into good sleep health (≥ 8 points) and poor sleep health (< 8 points). The diagnosis of OSA was based on a respiratory event index of ≥ 5.0 events per hour, with a predominance of obstructive respiratory events. Insomnia was characterized by the presence of one or more of the following nighttime complaints: difficulty initiating sleep, waking up during the night and having trouble falling back asleep, and waking up earlier than desired, occurring at least 3 times a week for 3 months, and causing daytime impairments

Figure 1 shows the distribution of individuals categorized as good sleepers or poor sleepers across four groups based on the diagnosis of OSA, insomnia, or COMISA, and those without any of these diagnoses. The percentage of poor sleepers was higher among those in the insomnia and COMISA groups (80.0% and 65.5%, respectively), whereas the control and OSA groups predominantly consisted of individuals classified as good sleepers (68.8% and 70.2%, respectively).

Table 3 presents the results of multiple logistic regression analyses examining several clinical and polysomnographic variables about poor sleep quality, as assessed by the Ru-SATED questionnaire. The logistic regression model showed satisfactory calibration, evaluated by the Hosmer–Lemeshow test, which resulted in a statistic of 2.629 (*p* = 0.955). In the univariate analysis, four factors were identified as statistically significant predictors of poor sleep health: the presence of EDS (*p* = 0.008), chronic insomnia (*p* < 0.001), ISI measurements (*p* < 0.001), and REI values (*p* = 0.020). However, in the multivariate analysis, only ISI values emerged as a statistically significant and independent predictor of poor sleep quality (*p* < 0.001).

**Table 3:** Univariate and multivariate tests for predicting a poor sleep quality (*n* = 415)

Parameters	Univariate analysis		Multivariate analysis	
	Crude OR (95% CI)	*p*-value	Adjusted OR (95% CI)	*p*-value
Female sex	1.183 (0.801–1.749)	0.398	-	-
Age, years	1.000 (0.989–1.012)	0.941	-	-
BMI, kg/m^2^	1.011 (0.982–1.041)	0.452	-	-
NC, cm	1.035 (0.994–1.077)	0.094	-	-
WC, cm	1.004 (0.992–1.016)	0.545	-	-
EDS	1.695 (1.149–2.501)	0.008	1.075 (0.672–1.719)	0.762
Hypertension	1.332 (0.905–1.960)	0.147	-	-
Type 2 DM	1.368 (0.883–2.117)	0.160	-	-
Dyslipidemia	1.174 (0.750–1.836)	0.483	-	-
Chronic insomnia	4.988 (3.280–7.583)	< 0.001	1.165 (0.658–2.052)	0.605
Current smoking	0.986 (0.468–2.077)	0.971	-	-
ISI, points	1.232 (1.180–1.287)	< 0.001	1.219 (1.154–1.287)	< 0.001
REI, n/h	1.013 (1.002–1.024)	0.020	1.004 (0.992–1.017)	0.503
T90, %	1.003 (0.995–1.011)	0.439	-	-
ODI, n/h	1.010 (0.998–1.023)	0.112	-	-
OSA	0.924 (0.588–1.452)	0.732	-	-

All estimates are reported as crude and adjusted odds ratios (ORs) with 95% confidence intervals (CIs). *BMI* body mass index; *NC* neck circumference; *WC* waist circumference; *DM* diabetes mellitus; *REI* respiratory event index; *T90* time with SpO_2_ < 90%; *ODI* oxygen desaturation index at 3%; *OSA* obstructive sleep apnea. Poor sleep quality (dependent variable) was characterized by the presence of Ru-SATED scores < 8 points

**Fig. 2:**
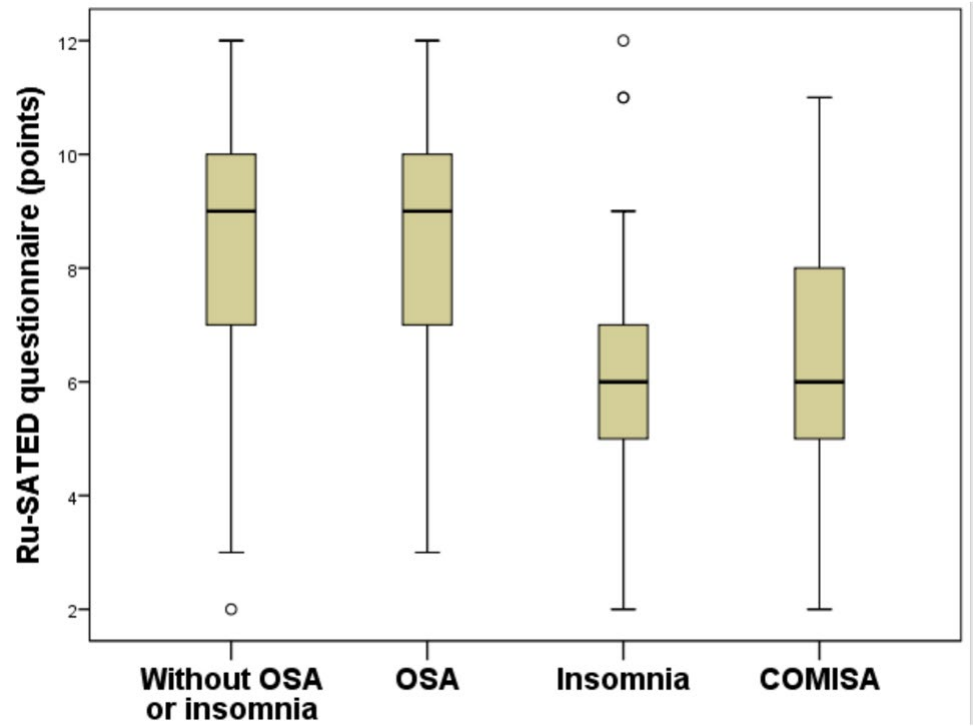
Box plot illustrating the distribution of final scores obtained from the Ru-SATED among 415 individuals divided into four groups: 64 subjects without insomnia and obstructive sleep apnea (OSA), 168 individuals with OSA alone, 35 with insomnia alone, and 148 with comorbid insomnia and OSA (COMISA). In the box plot, the box represents the 75th percentile (upper limit), the median (50th percentile), and the 25th percentile (lower limit). The lines extending from the box indicate the upper and lower limits of the distribution. The distribution of the final Ru-SATED scores showed significant differences among the four groups (*p* < 0.001). There were no significant differences in Ru-SATED scores between individuals without OSA and without insomnia (controls) and those with OSA alone (*p* = 0.951). No significant differences emerged between individuals with insomnia alone and those with COMISA (*p* = 0.735). OSA was diagnosed with a respiratory event index of ≥ 5.0 events per hour, primarily obstructive. Insomnia involved difficulties such as initiating sleep, waking during the night, or waking up too early, occurring at least three times a week for three months, causing daytime impairments

In our sample, the diagnosis of isolated OSA was unsurprisingly the most prevalent, accounting for 40.5% of participants. This was followed by individuals diagnosed with COMISA at 35.7%, those without either OSA or insomnia at 15.0%, and individuals with isolated insomnia at 8.4%. Figure 2 presents a box plot that represents the distribution of Ru-SATED questionnaire scores among the 415 study participants, categorized by the presence or absence of insomnia and OSA. The distribution of Ru-SATED scores showed statistically significant differences among the four groups: controls, individuals with only OSA, those with only insomnia, and those diagnosed with COMISA (*p* < 0.001). However, the median Ru-SATED scores were statistically similar between the control group and those with only OSA, both achieving a median score of 9.0 (IQR: 7.0–10.0; *p* = 0.951). Similarly, individuals with only insomnia had a median Ru-SATED of 6.0 points (IQR: 5.0–7.0), which was comparable to the median of those diagnosed with COMISA, who had a score of 6.0 points (IQR: 5.0–8.0; *p* = 0.735).

Multidimensional Ru-SATED scores were negatively correlated with the severity of chronic insomnia, as measured by the ISI (*r* = −0.625; *p* < 0.001). Conversely, there was no correlation between Ru-SATED scores and the REI (*r* = −0.076; *p* = 0.123), oxygen desaturation index (*r* = −0.081; *p* = 0.106), or percentage of time with blood oxygen saturation below 90% (*r* = −0.062; *p* = 0.215) obtained by HSAT. This indicates that Ru-SATED scores were significantly affected by the severity of chronic insomnia, but were not related to the severity of OSA, as assessed by the REI or by oximetry data.

## Discussion

Our study involved 415 outpatients with suspected SDB who underwent HSAT and Ru-SATED assessments. We found a high prevalence of poor sleep quality, particularly among those diagnosed with chronic insomnia, either alone or in conjunction with OSA (COMISA). In the univariate analysis, four factors—EDS, chronic insomnia, ISI, and REI—showed a significant association with poor sleep quality. However, only the severity of insomnia, as measured by ISI scores, was identified as a statistically significant and independent predictor of poor sleep quality in the cohort. In contrast, the presence or severity of SDB did not significantly affect sleep quality scores. To our knowledge, this is the first study designed to assess multidimensional sleep health using the Ru-SATED questionnaire in adult individuals with suspected SDB who were consecutively referred for an overnight sleep study.

The finding that the severity of chronic insomnia, rather than OSA, predicts poor sleep quality is in line with previous studies indicating that insomnia negatively affects individuals' subjective assessments of their sleep quality [18]. This has important practical implications, as it is common to find patients with symptoms of chronic insomnia even in centers that almost exclusively monitor individuals with SDB. Additionally, those diagnosed with COMISA often experience poorer subjective and objective sleep quality, reduced daytime functioning, and diminished mental health and quality of life [21–23].

However, most studies on sleep quality have primarily used the PSQI, which is less practical than the Ru-SATED questionnaire. In our analysis of the six categories of the Ru-SATED, we observed that lower scores were more prevalent among patients with poor sleep health compared to those with good sleep health. This trend was especially notable in the satisfaction (S) category of the questionnaire. In our sample, among individuals with poor sleep quality, almost 80% of patients reported that they were never or rarely satisfied with their sleep quality.

The PSQI is a widely validated tool that consists of 19 questions that assess seven components of sleep quality: 1) subjective sleep quality, 2) sleep latency, 3) sleep duration, 4) sleep efficiency, 5) sleep disturbances, 6) use of sleeping medications, and 7) daytime impacts [30]. Each component is scored on a 3-point scale, resulting in a total score of up to 21 points [30]. A PSQI score above 5 indicates poor sleep quality, while a score of 5 or below suggests good sleep quality [30]. Although the PSQI is a well-established instrument, it can be time-consuming to complete, which limits its routine use in clinical practice. In contrast, the Ru-SATED questionnaire offers a simpler format, making it more practical for routine evaluations of patients referred for evaluation of a sleep problem. The concept of sleep health was originally defined as a multidimensional pattern of sleep and wakefulness, tailored to meet individual, social, and environmental needs, while promoting both physical and mental well-being [8].

We included a relatively large sample of adults suspected of having OSA who were referred for HSAT. However, the study has some limitations that deserve mention. First, the study was conducted at a single center, which may limit the generalizability of our findings. All participants underwent HSAT on a single night, which could underestimate the true prevalence of OSA in our sample. We also did not collect data related to night-shift work, occupation type, marital status, or substance use, which are significant limitations. Additionally, due to the cross-sectional design of the study, we cannot establish any potential causal relationships.

## Conclusions

A high prevalence of poor sleep health was detected among individuals undergoing HSAT for suspected SDB. We found that the severity of chronic insomnia emerges as the only statistically significant and independent predictor of poor sleep quality among those individuals clinically suspected of suffering from OSA. Future studies should involve larger and more diverse populations, as well as a pre-post intervention design, to clarify the effects of treatment on sleep quality.

## Data Availability

The datasets generated during and/or analysed during the current study are available from the corresponding author on reasonable request.

## Abbreviations

BMI: Body mass index
CI: Confidence interval
COMISA: Comorbid insomnia and obstructive sleep apnea
EDS: Excessive daytime sleepiness
ESS: Epworth Sleepiness Scale
HSAT: Home sleep apnea test
IQR: Interquartile range
ISI: Insomnia Severity Index
OR: Odds ratio
OSA: Obstructive sleep apnea
PSQI: Pittsburgh Sleep Quality Index
REI: Respiratory event index
SDB: Sleep-disordered breathing
